# Impact of observation duration on behavioural pain assessment and intra-observer reliability in castrated piglets: A pilot study

**DOI:** 10.1017/awf.2025.22

**Published:** 2025-04-28

**Authors:** Rubia M Tomacheuski, Pedro HE Trindade, Victoria R Merenda, Magdiel Lopez-Soriano, Monique Pairis-Garcia

**Affiliations:** 1 Translational Research in Pain, Department of Clinical Sciences, College of Veterinary Medicine, North Carolina State University, Raleigh, NC, USA; 2 Department of Large Animal Clinical Sciences, College of Veterinary Medicine, Michigan State University, East Lansing, MI, USA; 3 Department of Population Health and Pathobiology, College of Veterinary Medicine, North Carolina State University, Raleigh, NC, USA

**Keywords:** animal welfare, castration, farm animal, pain measurement, procedural pain, swine

## Abstract

Pain monitoring and diagnosis are crucial in seeking to improve animal welfare. This pilot study aimed to investigate the impact of long hours observation on pain assessment and the intra-observer reliability in piglets using video recording. A total of ten piglets, five from the control group (sham castration; pain-free) and five from the pain group (surgical castration; pain-state), were video-recorded immediately post-castration. The videos were randomised and assessed by an experienced observer using the Unesp-Botucatu Pig Composite Acute Pain Scale (UPAPS). The same ten videos were watched at three different times (trial initiation, half-way point, trial termination) with a four-week interval between them. During the four-week interval periods, the observer watched an additional 360 videos from another study to simulate long observation periods. For the pain group, no differences were found in the *post hoc* test for the UPAPS total score, and most of the UPAPS items. In contrast, for the control group, the UPAPS total score was higher at the half-way time-point, and no differences were found between UPAPS items. The intra-class correlation coefficient (ICC) inferred ‘very good’ intra-observer reliability for UPAPS total score in all time-points of assessment for both groups. Video-recorded pain assessment is a reliable method to assess pain in piglets given that observation duration for pain assessment had only minimal impact on the UPAPS total score, and no differences were found among most of the items. From an animal welfare standpoint, video-recorded pain assessment is a non-invasive method, that can be an additional asset for pain research.

## Introduction

Pain assessment methods are used widely across veterinary medicine in diverse study designs to improve animal welfare by objectively assessing and diagnosing pain across multiple species (McLennan 2018; Vullo *et al.* 2020; Evangelista *et al.* 2021; Tomacheuski *et al.* 2023a). In 2023, the first behavioural pain assessment tool, the Unesp-Botucatu Pig Composite Acute Pain Scale (UPAPS), was validated for use in piglets through observable behaviours, using a scoring system to quantify pain levels, and a cut-off point for indication of analgesia (Robles *et al.* 2023). This is a critically important tool to evaluate the effect of invasive procedures performed in livestock and for the US swine industry as it can be used in various settings by pharmaceutical companies to validate analgesic drug efficacy in swine and pursue product approval through the US Food and Drug Administration (Wagner *et al.* 2020).

Video-recorded pain assessment is a non-invasive method to observe and assess behaviour where animals are filmed at different time-points, and videos are later assessed by observers (Brondani *et al.* 2011; Oliveira *et al.* 2014; Luna *et al.* 2020; Silva *et al.* 2020; Belli *et al.* 2021; Haddad Pinho *et al.* 2022). There are advantages to the video-recorded pain assessment as it can reduce bias from human-animal interaction, it can be rewatched multiple times and can be checked for reliability (Mokkink *et al.* 2018, 2020; Tomacheuski *et al.* 2023a). However, assessing pain via pre-recorded video is laborious and requires long observation periods, and there are limitations of observing pain on video, which requires picking up subtle behaviours and therefore to be highly attentive, compared to when observing simpler behaviours such as location or posture (Robles *et al.* 2023; Tomacheuski *et al.* 2023b). For example, in a recent study of pain assessment in cattle (Tomacheuski *et al.* 2023b), researchers observed over 4 h of video (95 videos) for one project over a period of one month, not taking into account additional time needed to input data or rewatch video segments. To date, there have been no studies evaluating whether observation duration influences the reliability and consistency of pain assessment in swine using pre-recorded videos. The aim of this study therefore was to investigate the impact of observation duration on pain assessment and the intra-observer reliability in castrated piglets using video recording.

## Materials and methods

### Ethical approval

This study was approved by the Institutional Animal Care and Use Committee of North Carolina State University (IACUC protocol: 20-113-01). In the US, piglet castration without anaesthetic or analgesia is permitted as part of standard production practices, with no legislation mandating pain relief. The American Veterinary Medical Association (AVMA) recommends performing the procedure between 2 and 14 days of age (AVMA 2025). This study was part of a larger experiment that was conducted by Lopez-Soriano *et al.* (2022) from January to March 2021 at a commercial swine breeding facility located in the Southeastern United States.

### Study animals

A subset of ten Large White × Duroc cross male piglets enrolled in a larger study conducted previously (Lopez-Soriano *et al.* 2022) were selected to be included for this dataset. Ten piglets, from different litters, between 2–8 days (± 4 days) of age, were housed with sows on fully slatted and tunnel-ventilated farrowing rooms and randomly assigned to one of two groups: (1) Pain (n = 5 piglets surgically castrated; pain state); or (2) Control (n = 5 piglets handled but not castrated; pain-free).

### Surgical procedure

The procedure was executed by a caretaker who had over ten years of experience performing castration. The piglets enrolled in the Pain group were picked up from the farrowing crate, held individually on the experimenter’s lap, positioned in dorsal recumbency, and subjected to two vertical incisions using a scalpel blade. Following the incision, the testicles became visible, at which point the spermatic cords were incised and the testicles fully extracted through traction. After the procedure they were returned to the farrowing crate. To replicate similar handling circumstances, a simulated castration procedure was performed on the Control group. This involved lifting the piglets, holding them individually, placing them in dorsal recumbency, and the same caretaker responsible for the surgical castration applying pressure to the scrotal region (Lopez-Soriano *et al.* 2022).

### Observer training

The observer (RMT) has experience with farm animal pain assessment and underwent training previously to learn how to assess pain using the Unesp-Botucatu Pig Composite Acute Pain Scale (UPAPS; Table S1 [see Supplementary material]) for piglets. The UPAPS is a species-specific behavioural scale that quantifies pain, and presents a cut-off point for indicating the need for analgesic intervention (https://animalpain.org/en/porcos-dor-en/). Two training sessions were conducted during which the observer participated in a 2-h presentation on the pain scale that included video examples for each pain scale item. Following this training, RMT watched and scored ten videos (not part of this study) over a period of a week and scores were compared to an experienced researcher in pig pain assessment (MLS) for inter-reliability using inter-class correlation coefficient (ICC). The observer was permitted to continue with the study if an ICC ≥ 0.90 was achieved. The observer achieved an ICC of 0.93 after the training.

### Video recording and pain assessment

**Figure 1.**
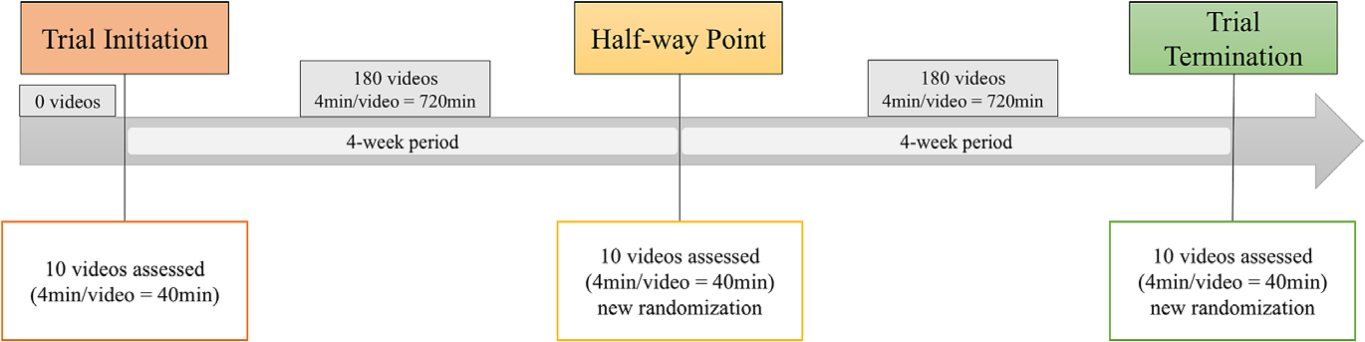
Timeline of video-recorded assessment of piglets using the Unesp-Botucatu Pig Composite Acute Pain Scale (UPAPS).

The animals were recorded using high-definition cameras (Sony HDR-CX405; New York, NY, USA), installed to the back of the farrowing crate using zip ties, angled at 45° facing the heating mat. One camera was used per crate. To determine the video observation duration effect over time, RMT completed two behavioural datasets. RMT was masked to the procedure and groups. The first dataset included ten videos of pain and pain-free piglets previously described in the methodology. Each video had a duration of 4 min, totalling 40 min of video-recordings. These videos were all observed within a day, at three time-points: trial initiation; half-way point; and trial termination, each separated by a four-week interval. At each time-point, the videos were randomised differently. A second dataset comprised of 360 videos (4 min per video = 1,440 min) from an external swine castration project was used to mirror long observation periods, those videos were observed to assess pain using UPAPS. Following video observation of the ten videos at trial initiation, 180 videos were observed over a four-week period. The ten videos from the first dataset were then re-watched at the half-way point and the remaining 180 videos were observed over a four-week period for a total of 1,560 min of behavioural assessment (Figure 1).

### Statistical analysis

Statistical analyses were performed in R software, using the integrated development environment RStudio (Version 4.1.0; 2021-06-29; RStudio Inc). The functions and packages used are presented in the format ‘package::function’ corresponding to the computational programming language in R. A significance level of *P* ≤ 0.05 and tendency of 0.05 < *P* ≤ 0.10 were considered for all tests. All figures were built with a palette of colours readily distinguishable by people with common forms of colour blindness (ggplot2::scale_colour_viridis_d).

Modelling was conducted to analyse the consistency among the three time-points of assessment. Then, a multilevel negative binomial model (lme4::glmer.nb) was conducted using UPAPS total score as the response variable. Bonferroni was used for adjusting the multiple comparisons in the *post hoc* test (lsmeans::lsmeans and multcomp::cld). An additional model was built after deleting outliers (i.e. observations with UPAPS total score higher than average plus three standard deviations) for each time-point in each group.

The intra-class correlation coefficient (ICC), two-way random effects model, agreement type among single observer and measurements, and its 95% confidence interval (irr::icc) were used to evaluate the intra-observer reliability for all variables. The interaction between time-points and treatment groups was used as an explanatory variable and video identification was included as random effect. The interpretation of ICC was ‘very good’ 0.81–1.0; ‘good’ 0.61–0.80; ‘moderate’ 0.41–0.60; ‘reasonable’ 0.21–0.4; and ‘poor’ ≤ 0.2 (Altman 1991). The dataset can be found as part of the Supplementary material.

## Results

**Figure 2.**
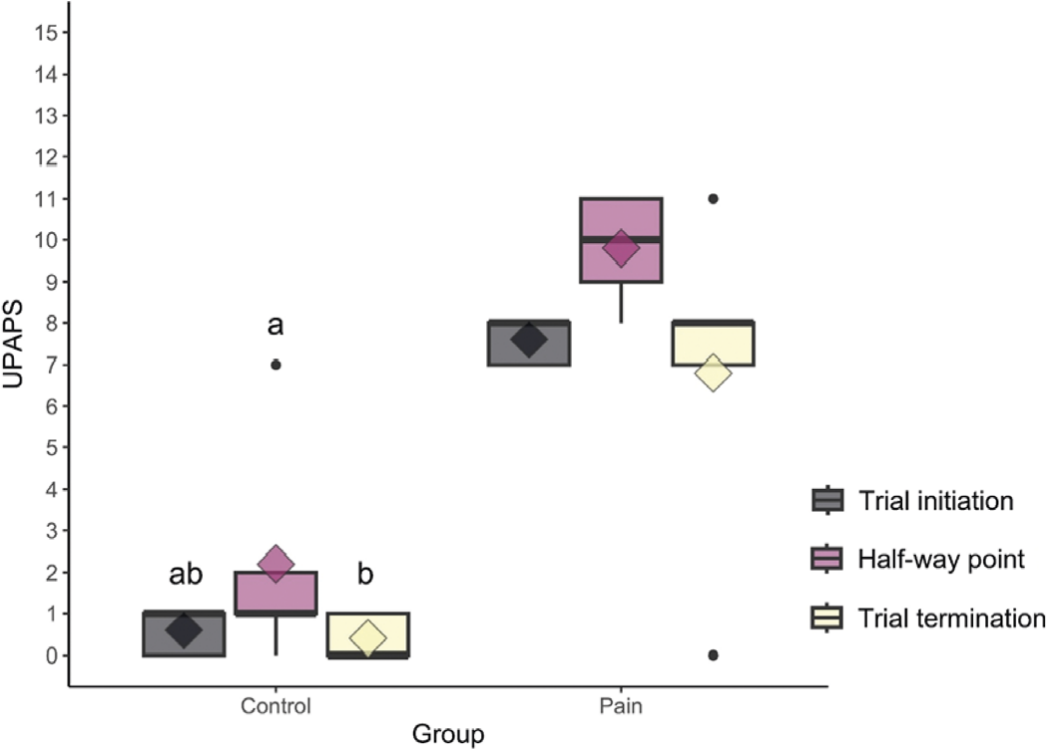
Boxplots of UPAPS total score over the three time-points of assessment for control and pain groups of ten piglets over time (trial initiation, half-way point, and trial termination). UPAPS is the Unesp-Botucatu Pig Composite Acute Pain Scale; the black circles indicate outliers; the diamond indicates the mean; the top and bottom box lines represent the interquartile range (25 to 75%); the bold line within the box represents the median; lowercase letters indicate a significant difference between time-points for each treatment group.

For the pain group, no differences were found in the *post hoc* test for the UPAPS total score, and most of the UPAPS items. However, UPAPS total scores were higher at the half-way point than trial termination time-point for the control group (Figure 2). The UPAPS total scores without the outliers can be found in Figure S1 (see Supplementary material), and for each video and each assessment over time-points in Figure S2 (as above).

The UPAPS total score, items ‘posture’, ‘interaction and interest in the surroundings’, and subitem ‘the head is below the line of the spinal column’ had very good intra-observer reliability over the three time-points of video-recorded assessment (Table 1). The other UPAPS items ‘activity’, ‘attention to affected area’ and ‘miscellaneous behaviours’ varied from very good to moderate intra-observer reliability (Table 1).

**Table 1.** Intra-observer reliability and 95% confidence interval of items, subitems, and UPAPS total score over the three time-points of video assessment of ten piglets

Items and subitems		Trial Initiation vs Half-way Point			Trial Initiation vs Trial Termination			Half-way Point vs Trial Termination		
		ICC	Lower	Upper	ICC	Lower	Upper	ICC	Lower	Upper
Posture		**0.97**	0.90	0.99	**0.99**	0.95	1.00	**0.96**	0.84	0.99
Interaction and interest in the surroundings		**0.96**	0.85	0.99	**0.90**	0.61	0.98	**0.89**	0.55	0.97
Activity		0.50	–1.02	0.88	0.80	0.18	0.95	0.72	–0.15	0.93
Attention to affected area		0.72	–0.14	0.93	0.53	–0.89	0.88	**0.92**	0.69	0.98
	A. Elevates pelvic limb or alternates the support of the pelvic limb	NA	NA	NA	NA	NA	NA	NA	NA	NA
	B. Scratches or rubs the painful area	**0.86**	0.44	0.97	0.55	–0.83	0.89	0.36	–1.59	0.84
	C. Moves and/or runs away and/or jumps after injury of the affected area	NA	NA	NA	NA	NA	NA	0.64	–0.46	0.91
	D. Sits with difficulty	0.66	–0.39	0.91	0.66	–0.39	0.91	**1.00**	1.00	1.00
Miscellaneous behaviours		0.49	–1.04	0.87	**0.86**	0.43	0.96	NA	NA	NA
	A. Wags tail continuously and intensely	0.78	0.12	0.95	0.78	0.12	0.95	–0.25	–4.03	0.69
	B. Bites the bars or objects	NA	NA	NA	NA	NA	NA	0.64	–0.46	0.91
	C. The head is below the line of the spinal column	**0.90**	0.59	0.97	**1.00**	1.00	1.00	**0.90**	0.59	0.97
	D. Presents difficulty in overcoming obstacles	NA	NA	NA	NA	NA	NA	NA	NA	NA
UPAPS total score		**0.94**	0.76	0.99	**0.88**	0.51	0.97	**0.88**	0.51	0.97

NA represents “not available” when the number of observations was insufficient between the timepoints; ICC, intraclass correlation coefficient. Bold results represent ‘very good’ ICC. The ICC was defined as ‘very good’ 0.81–1.0; ‘good’ 0.61–0.80; ‘moderate’ 0.41–0.60; ‘reasonable’ 0.21–0.4; or ‘poor’ ≤ 0.2.

Table 2 depicts the mean score for each item and subitem of UPAPS when analysing the pain group. Overall, the means for each item and subitem did not differ between time-points of assessment. However, the mean UPAPS score for ‘attention to affected area’ was higher at the half-way point than at the trial initiation time-point (Table 2).

**Table 2.** Mean (± SD) of items, sub-items, and UPAPS (Unesp-Botucatu Pig Composite Acute Pain Scale) total score over three time-points of assessment of piglets in the pain group (n = 5)

Items and subitems		Time-points		
		Trial Initiation	Half-way Point	Trial Termination
Posture		3.00 (± 0.00)	3.00 (± 0.00)	2.80 (± 0.45)
Interaction and interest in the surroundings		1.80 (± 0.45)	2.20 (± 0.45)	1.60 (± 0.55)
Activity		1.00 (± 0.00)	1.80 (± 1.10)	1.40 (± 0.89)
**Attention to affected area**		**0.60 (± 0.55)**^**b**^	**1.8(± 0.45)**^**a**^	**1.60 (± 0.55)**^**ab**^
	A. Elevates pelvic limb or alternates the support of the pelvic limb	0.00 (± 0.00)	0.00 (± 0.00)	0.00 (± 0.00)
	B. Scratches or rubs the painful area	0.20 (± 0.45)	0.20 (± 0.45)	0.40 (± 0.55)
	C. Moves and/or runs away and/or jumps after injury of the affected area	0.00 (± 0.00)	0.60 (± 0.55)	0.20 (± 0.45)
	D. Sits with difficulty	0.40 (± 0.55)	1.00 (± 0.00)	1.00 (± 0.00)
Miscellaneous behaviours		1.20 (± 0.45)	1.00 (± 0.00)	1.40 (± 0.89)
	A. Wags tail continuously and intensely	0.20 (± 0.45)	0.00 (± 0.00)	0.20 (± 0.45)
	B. Bites the bars or objects	0.00 (± 0.00)	0.00 (± 0.00)	0.00 (± 0.00)
	C. The head is below the line of the spinal column	1.00 (± 0.00)	1.00 (± 0.00)	1.00 (± 0.00)
	D. Presents difficulty in overcoming obstacles	0.00 (± 0.00)	0.00 (± 0.00)	0.20 (± 0.45)
UPAPS total score		7.60 (± 0.55)	9.80 (± 1.30)	6.80 (± 4.09)

Bold highlight *P* ≤ 0.05.

Table 3 depicts the mean score for each item and subitem of UPAPS when analysing the control group. Overall, the means for each item and subitem did not differ between time-points of assessment. However, the mean UPAPS total score was higher at the half-way point than at the trial termination time-point (Table 3).

**Table 3.** Mean (± SD) of items, sub-items, and UPAPS (Unesp-Botucatu Pig Composite Acute Pain Scale) total score over three timepoints of assessment of piglets in the control group (n = 5)

Items and subitems		Time-points		
		Trial Initiation	Half-way Point	Trial Termination
Posture		0.20 (± 0.45)	0.20 (± 0.45)	0.20 (± 0.45)
Interaction and interest in the surroundings		0.00 (± 0.00)	0.00 (± 0.00)	0.00 (± 0.00)
Activity		0.00 (± 0.00)	0.60 (± 1.34)	0.00 (± 0.00)
Attention to affected area		0.20 (± 0.45)	0.40 (± 0.55)	0.00 (± 0.00)
	A. Elevates pelvic limb or alternates the support of the pelvic limb	0.00 (± 0.00)	0.00 (± 0.00)	0.00 (± 0.00)
	B. Scratches or rubs the painful area	0.20 (± 0.45)	0.40 (± 0.55)	0.00 (± 0.00)
	C. Moves and/or runs away and/or jumps after injury of the affected area	0.00 (± 0.00)	0.00 (± 0.00)	0.00 (± 0.00)
	D. Sits with difficulty	0.00 (± 0.00)	0.00 (± 0.00)	0.00 (± 0.00)
Miscellaneous behaviours		0.20 (± 0.45)	1.00 (± 0.71)	0.20 (± 0.45)
	A. Wags tail continuously and intensely	0.20 (± 0.45)	0.20 (± 0.45)	0.00 (± 0.00)
	B. Bites the bars or objects	0.00 (± 0.00)	0.60 (± 0.55)	0.20 (± 0.45)
	C. The head is below the line of the spinal column	0.00 (± 0.00)	0.20 (± 0.45)	0.00 (± 0.00)
	D. Presents difficulty in overcoming obstacles	0.00 (± 0.00)	0.00 (± 0.00)	0.00 (± 0.00)
**UPAPS total score**		**0.60 (± 0.55)**^**ab**^	**2.20 (± 2.77)**^**a**^	**0.40 (± 0.55)**^**b**^

Bold signifies: *P* ≤ 0.05.

## Discussion

Pain assessment is a critical aspect for safeguarding on-farm pig welfare as it allows for timely intervention and appropriate pain management for routine production procedures. This is the first study to investigate the impact of long hours observation duration on video-recorded assessment. The original study which developed the UPAPS (Luna *et al.* 2020) also evaluated 180 videos in total for its validation, but the impact of the long hours was not investigated. This study demonstrated minimal impact of the observation duration on video-recorded pain assessment method and highlights the importance of assessing reliability over time.

As expected, the UPAPS scores were higher for the pain group than the control group over the three time-points of assessment. While no differences were found to the pain group UPAPS total score even considering the two outliers at the trial termination time-point, and among the items, only the ‘attention to affected area’ inferred difference between time-points. Hence, in the control group, the scores for UPAPS at the half-way point of assessment were higher than at the trial termination time-point, but no differences were found for UPAPS items. The difference found at the half-way point could be related to the outlier present in the assessment. Those results infer that the long hours of observation have impacted the second time-point of assessment for the control group (pain-free piglets), but not the pain group videos. This, despite one of the outliers from the pain group at trial termination being scored zero, perhaps as a result of workload and observer fatigue, and the degree of subjectivity of the instrument. One hypothesis is that the sheer quantity of videos (180 = 1,560 min) generate visual fatigue over four weeks of assessment and decrease the observer’s attention. Moreover, there is no literature investigating this. Another hypothesis is that other variables, such as emotional status, attention to detail, motivation, course workload, could have impacted the observer’s attention. Future studies should further research the possible reasons for this impact, recording the subjective variables (e.g. emotional status, attention, etc), adding more observers, with different levels of experience, and testing whether smaller quantities of videos (50, 100, 120) would give rise to a different outcome.

Intra-observer reliability (i.e. repeatability) is an important analysis to infer if an instrument is reliable (Streiner *et al.* 2015; Tomacheuski *et al.* 2023a). The ICC demonstrated ‘very good’ results to items ‘posture’, and ‘interaction and interest in the surroundings’, and UPAPS total score. Those results were superior to the first study that developed and validated the UPAPS since their ICC for ‘posture’, ‘interaction’, and UPAPS total score ranged from 0.88 to 0.64 (Luna *et al.* 2020). Moreover, this affirms the clinical value of pain assessment videos as reliable instruments in piglets. We would recommend that observers who evaluate a substantial number of videos conduct intra-observer reliability assessments throughout different times during the entire observation period. This helps ensure behaviours are recorded in a consistent, reliable and uniform manner.

This pilot study has limitations, the number of observers should be increased for future studies, with both male and female observers included in the group (Tomacheuski *et al.* 2023a). This study was conducted with a model of castration, future studies should be performed with other pain models, and in a real-time observation clinical setting. Despite this study having been conducted with a limited number of videos (n = 10), it still found significant differences; future studies should increase the sample size, to confirm these outcomes. Additionally, the same ten videos were rewatched three times which could have generated a bias of recall for the observer, although the videos were recorded and animals kept in groups with the sows in similar conditions as the 360 videos watched, it would make it harder for the observer to differentiate them. Future studies should randomise the videos among the main study to avoid recall bias. Another limitation is that the subjective variables (e.g. fatigue, emotional status, attention to detail, motivation, course workload, etc) were not recorded and their impact on video assessment were not analysed. This should be further investigated.

While several studies have used long hours observation duration to research pain (Brondani *et al.* 2011; Oliveira *et al.* 2014; Luna *et al.* 2020; Silva *et al.* 2020; Belli *et al.* 2021; Haddad Pinho *et al.* 2022), as yet none had ever investigated the impact of observation duration on pain assessments. As a practical implication, this pilot study demonstrated a minimal impact from the long hours video assessment, and those results question how many hours should represent the ideal approach for video-assessment in clinical and experimental research. Future studies should investigate the optimal daily hours of video assessment to mitigate the impact on research.

### Animal welfare implications

From an animal welfare standpoint, video-recorded assessment represents a non-invasive, reliable and convenient method that may be used for pain research, increasing the accuracy of pain assessment evaluations and consequently improving the animal welfare of pigs. Given that assessing pain via video recordings does not rely upon handling or interacting with the animals, it is a tool that may be used even when labour is scarce or in research trials that may be compromised by additional handling of the animals.

## Conclusion

Video-recorded pain assessment is a reliable method for assessing pain in piglets given that observation duration for pain assessment had only minimal impact on the UPAPS total score and no differences were found among most of the items. Highly encouraging as regards the reliability of video-recorded pain assessment for experimental and clinical trials in piglets undergoing painful procedures and the importance of carrying out intra-observer reliability during larger studies. Future studies should be carried to confirm these findings.

## Supporting information

Tomacheuski et al. supplementary material 1 Tomacheuski et al. supplementary material

Tomacheuski et al. supplementary material 2 Tomacheuski et al. supplementary material

## Supplementary material

The supplementary material for this article can be found at https://doi.org/10.1017/awf.2025.22.

## References

[r1] Altman DG 1991 Practical Statistics for Medical Research, First Edition. Chapman and Hall: London, UK.

[r2] AVMA 2025 *Swine castration.* American Veterinary Medical Association. https://www.avma.org/resources-tools/avma-policies/swine-castration (accessed 5 March 2025).

[r3] Belli M, de Oliveira AR, de Lima MT, Trindade PHE, Steagall PV and Luna SPL 2021 Clinical validation of the short and long UNESP-Botucatu scales for feline pain assessment. PeerJ 9: e11225. 10.7717/PEERJ.11225/SUPP-4 33954046 PMC8048399

[r4] Brondani JT, Luna SPL and Padovani CR 2011 Refinement and initial validation of a multidimensional composite scale for use in assessing acute postoperative pain in cats. American Journal of Veterinary Research 72: 174–183. 10.2460/ajvr.72.2.174 21281191

[r5] Evangelista MC, Monteiro BP and Steagall P V 2021 Measurement properties of grimace scales for pain assessment in non-human mammals: a systematic review. Pain 163: e697–e714. 10.1097/j.pain.0000000000002474 34510132

[r6] Haddad Pinho R, Luna SPL, Esteves Trindade PH, Augusto Justo A, Santilli Cima D, Werneck Fonseca M, Watanabe Minto B, Del Lama Rocha F, Miller A, Flecknell P and Leach MC 2022 Validation of the rabbit pain behaviour scale (RPBS) to assess acute postoperative pain in rabbits (*Oryctolagus cuniculus*). PLoS One 17: e0268973. 10.1371/journal.pone.0268973 35617348 PMC9135295

[r7] Lopez-Soriano M, Rocha Merenda V, Esteves Trindade PH, Loureiro Luna SP and Pairis-Garcia MD 2022 Efficacy of transdermal flunixin in mitigating castration pain in piglets. Frontiers in Pain Research 3. 10.3389/fpain.2022.1056492 PMC9691683 36438445

[r8] Luna SPL, Araújo AL de, Neto PI da N, Brondani JT, Oliveira FA de, Azerêdo LM dos S, Telles FG and Trindade PHE 2020 Validation of the UNESP-Botucatu pig composite acute pain scale (UPAPS). PLoS One 15: e0233552. 10.1371/journal.pone.0233552 32480399 PMC7263847

[r9] McLennan KM 2018 Why pain is still a welfare issue for farm animals, and how facial expression could be the answer. Agriculture (Switzerland) 8: 127. 10.3390/agriculture8080127

[r11] Mokkink LB, Boers M, Vleuten CPM van der, Bouter LM, Alonso J, Patrick DL, Vet HCW de and Terwee CB 2020 COSMIN Risk of Bias tool to assess the quality of studies on reliability or measurement error of outcome measurement instruments: a Delphi study. BMC Medical Research Methodology: 1–26. 10.21203/rs.3.rs-40864/v2 PMC7712525 33267819

[r12] Mokkink LB, Prinsen CAC, Patrick DL, Alonso J, Bouter LM, De Vet HCW and Terwee CB 2018 COSMIN methodology for systematic reviews of Patient ‐ Reported Outcome Measures (PROMs). User Manual. Quality of Life Research: 1–78. 10.1007/s11136-018-1798-3 PMC5891568 29435801

[r13] Oliveira FA, Luna SPL, Amaral JB, Rodrigues KA, Sant’Anna AC, Daolio M and Brondani JT 2014 Validation of the UNESP-Botucatu unidimensional composite pain scale for assessing postoperative pain in cattle. BMC Veterinary Research 10: 200. 10.1186/s12917-014-0200-0 25192598 PMC4172785

[r14] Robles I, Luna SPL, Trindade PHE, Lopez-Soriano M, Merenda VR, Viscardi AV, Tamminga E, Lou ME and Pairis-Garcia MD 2023 Validation of the Unesp-Botucatu pig composite acute pain scale (UPAPS) inpiglets undergoing castration. PLoS One 18: e0284218. 10.1371/journal.pone.0284218 37053294 PMC10101451

[r15] Silva NEOF, Trindade PHE, Oliveira AR, Taffarel MO, Moreira MAP, Denadai R, Rocha PB and Luna SPL 2020 Validation of the Unesp-Botucatu composite scale to assess acute postoperative abdominal pain in sheep (USAPS). PLoS One 15: e0239622. 10.1371/journal.pone.0239622 33052903 PMC7556455

[r16] Streiner DLD, Norman GR and Cairney J 2015 Health Measurement Scales: a practical guide to their development and use. In: Streiner DLD, Norman GR and Cairney J (eds) *New York: Oxford University Press Inc*, *Fifth Edition*. Oxford University Press: Oxford, UK.

[r17] Tomacheuski RM, Monteiro BP, Evangelista MC, Luna SPL and Steagall PV 2023a Measurement properties of pain scoring instruments in farm animals: A systematic review using the COSMIN checklist. PLoS One 18: e0280830. 10.1371/JOURNAL.PONE.0280830 36662813 PMC9858734

[r18] Tomacheuski RM, Oliveira AR, Trindade PHE, Oliveira FA, Candido CP, Teixeira Neto FJ, Steagall PV and Luna SPL 2023b Reliability and validity of UNESP-Botucatu Cattle Pain Scale and Cow Pain Scale in *Bos taurus* and *Bos indicus* bulls to assess postoperative pain of surgical orchiectomy. Animals 13: 364. 10.3390/ani13030364 36766253 PMC9913732

[r19] Vullo C, Barbieri S, Catone G, Graic JM, Magaletti M, Di Rosa A, Motta A, Tremolada C, Canali E and Dalla Costa E 2020 Is the Piglet Grimace Scale (PGS) a useful welfare indicator to assess pain after cryptorchidectomy in growing pigs? Animals 10. 10.3390/ani10030412 PMC7143901 32131424

[r20] Wagner B, Royal K, Park R and Pairis-Garcia M 2020 Identifying barriers to implementing pain management for piglet castration: A focus group of swine veterinarians. Animals 10: 1202. 10.3390/ANI10071202 32679777 PMC7401590

