# Supplementary material for: Impact of observation duration on behavioural pain assessment and intra-observer reliability in castrated piglets: A pilot study
Source: Anim Welf. 2025 Apr 28;34:e27. doi: 10.1017/awf.2025.22 (PMC12056411; doi:10.1017/awf.2025.22)
Supplement: Tomacheuski et al. supplementary material 2 — Tomacheuski et al. supplementary material [file S0962728625000223sup002.pdf]

Impact of observation duration on behavioural pain assessment and intra-observer reliability in castrated piglets: A pilot study

Rubia M Tomacheuskihttps://orcid.org/0000-0002-7967-0124[1], Pedro HE Trindade[2], Victoria R Merenda[3], Magdiel Lopez-Soriano[3], Monique Pairis-Garcia[3]

[1]Translational Research in Pain, Department of Clinical Sciences, College of Veterinary Medicine, North Carolina State University, Raleigh, NC, USA

[2]Department of Large Animal Clinical Sciences, College of Veterinary Medicine, Michigan State University, East Lansing, MI, USA

[3]Department of Population Health and Pathobiology, College of Veterinary Medicine, North Carolina State University, Raleigh, NC, USA

Author for correspondence: Monique Pairis-Garcia, email: pairis-garcia@ncsu.edu

Supplementary material

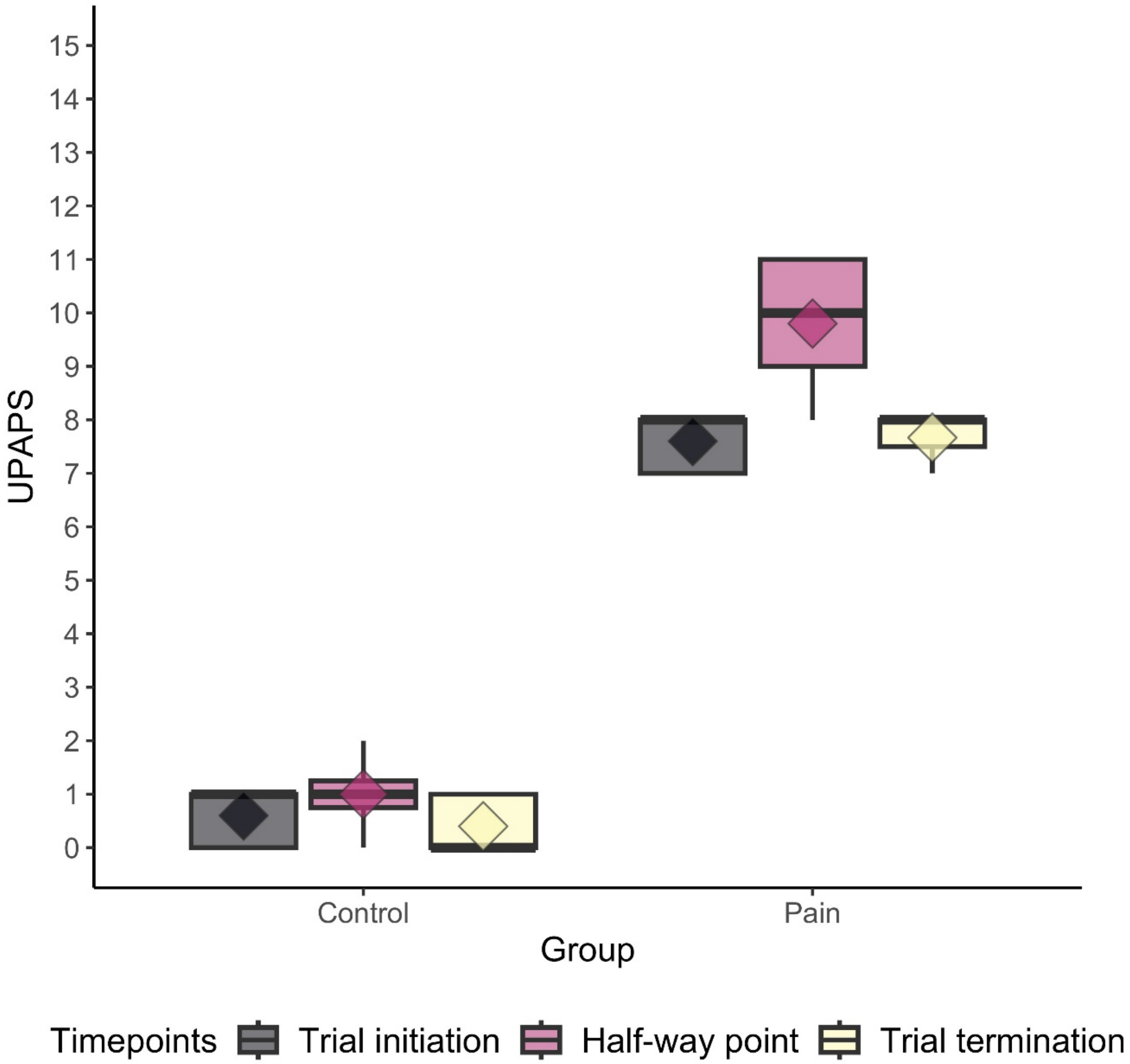

Figure S1. Boxplots of UPAPS total score without outliers, over the three time-points of video assessment (trial initiation, half-way point, and trial termination) for control and pain groups of piglets (UPAPS is the Unesp-Botucatu Pig Composite Acute Pain Scale; the diamond indicates the mean; the top and bottom box lines represent the interquartile range (25 to 75%); the bold line within the box represents the median)

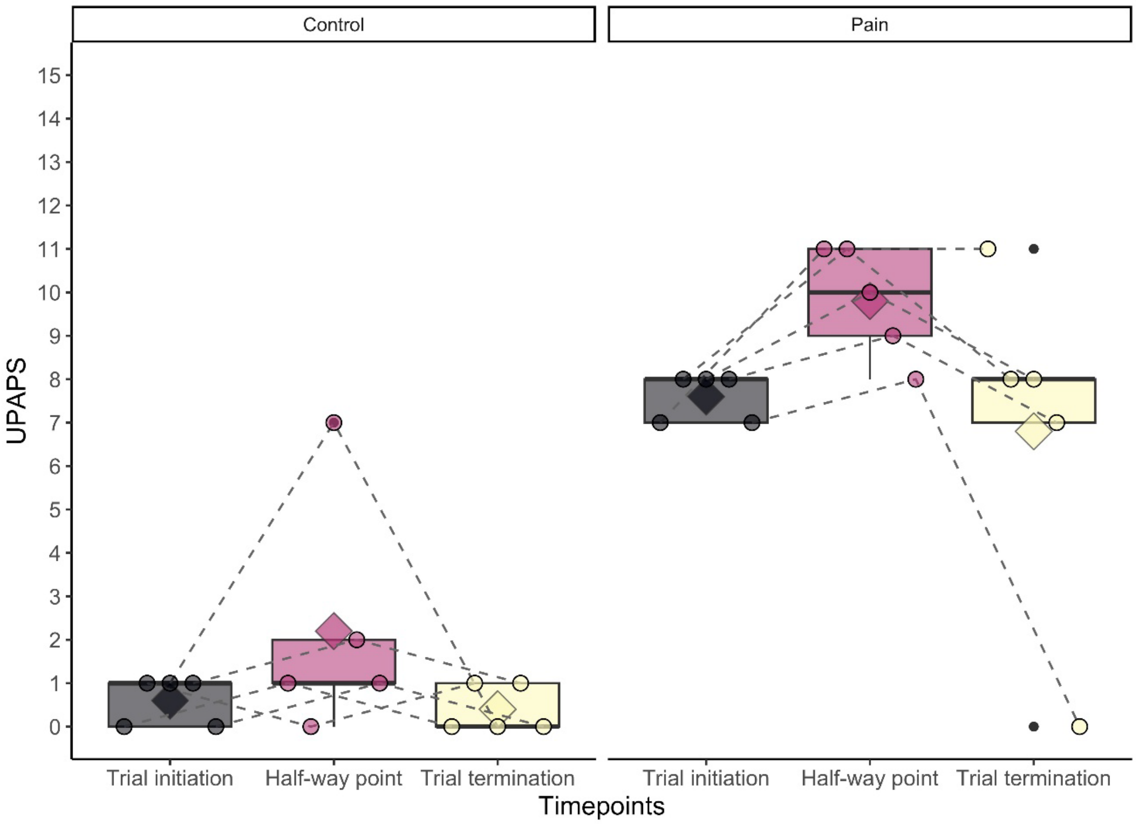

Figure S2. Boxplots of UPAPS total score over the three time-points of video assessment (trial initiation, half-way point, and trial termination) for control and pain groups of piglets (UPAPS is the Unesp-Botucatu Pig Composite Acute Pain Scale; each circle indicates each video; the diamond indicates the mean; the black circles indicate outliers; the top and bottom box lines represent the interquartile range (25 to 75%); the bold line within the box represents the median; and the dashed line indicates the dynamic of each video over time-points).

Table S1. Unesp-Botucatu pig composite acute pain scale (UPAPS)

Item	Score	Score/criterion	Links to videos
Posture	0	Normal (any position, apparent comfort, relaxed muscles) or sleeping	https://youtu.be/QSosCD2SD4E
	1	Changes posture, with discomfort	https://youtu.be/SpaWsFCrPxE
	2	Changes posture, with discomfort, and protects the affected area	https://youtu.be/VjSlsRrG8yA
	3	Quiet, tense, and back arched	https://youtu.be/pm4hJ5163ao
Interaction and interest in the surroundings	0	Interacts with other animals; interested in the surroundings or sleeping	https://youtu.be/-880STgYq2I
	1	Only interacts if stimulated by other animals; interested in the surroundings.	https://youtu.be/nXjOdwn3dyw
	2	Occasionally moves away from the other animals, but accepts approaches; shows little interest in the surroundings	https://youtu.be/2k2JDr5U6As
	3	Moves or runs away from other animals and does not allow approaches; disinterested in the surroundings	https://youtu.be/se70oYXcWFw
Activity	0	Moves normally or sleeping	https://youtu.be/cC75t7L5-YA
	1	Moves with less frequency	https://youtu.be/lQo9wq8LAn8
	2	Moves constantly, restless	https://youtu.be/YQRJjijLvpk
	3	Reluctant to move or does not move	https://youtu.be/Zyx0G3Wpt8o
Attention to the affected area		A. Elevates pelvic limb or alternates the support of the pelvic limb	https://youtu.be/UD99ftO7HE0
		B. Scratches or rubs the painful area	https://youtu.be/7idfFk1harE
		C. Moves and/or runs away and/or jumps after injury of the affected area	https://youtu.be/u-Pqubom278
		D. Sits with difficulty	https://youtu.be/ETNEOCVV4h0
	0	All the above behaviours are absent	
	1	Presence of one of the above behaviours	
	2	Presence of two of the above behaviours	
	3	Presence of three or all the above behaviours	
Miscellaneous behaviours		A. Wags tail continuously and intensely	https://youtu.be/pU5dGZFNRHc
		B. Bites the bars or objects	https://youtu.be/cF3dsq7gMtk
		C. The head is below the line of the spinal column.	https://youtu.be/ZcIgngclRpI
		D. Presents difficulty in overcoming obstacles (example: another animal)	https://youtu.be/HlvdOI3lGuY
	0	All the above behaviours are absent	
	1	Presence of one of the above behaviours	
	2	Presence of two of the above behaviours	
	3	Presence of three or all the above behaviours	
UPAPS total score	0-15	Pain intensity is represented by the sum of all behaviours scores	
